# CDC Grand Rounds: Reducing Severe Traumatic Brain Injury in the United States

**Published:** 2013-07-12

**Authors:** David W. Wright, Arthur Kellermann, Lisa C. McGuire, Bin Chen, Tanja Popovic

**Affiliations:** Emergency Neurosciences, Emory Univ School of Medicine, Atlanta, Georgia; RAND Health, RAND Corporation, Santa Monica, California; Div of Unintentional Injury Prevention, National Center for Injury Prevention and Control; Div of Laboratory Science and Standards, Laboratory Science, Policy, and Practice Program Office, Office of Surveillance, Epidemiology, and Laboratory Services; Office of the Director, CDC

A traumatic brain injury (TBI) is caused by a bump, blow, jolt, or penetrating wound to the head that disrupts the normal functioning of the brain (1). In 2009, CDC estimated that at least 2.4 million emergency department visits, hospitalizations, or deaths were related to a TBI, either alone or in combination with other injuries (2). Approximately 75% of TBIs are mild, often called concussions (3). Children, adolescents, and older adults are most likely to sustain a TBI (4). Nearly one third (30.5%) of all injury deaths included a diagnosis of TBI (5). In addition, an estimated 5.3 million U.S. residents are living with TBI-related disabilities, including long-term cognitive and psychologic impairments (6). A severe TBI not only affects a person’s life and family, but also has a large societal and economic toll. The economic costs of TBIs in 2010 were estimated at $76.5 billion, including $11.5 billion in direct medical costs and $64.8 billion in indirect costs (e.g., lost wages, lost productivity, and nonmedical expenditures) (7,8). These data underestimate the national burden because they include neither TBIs managed in nonhospital settings nor >31,000 military personnel diagnosed with TBI and treated in the U.S. Department of Defense or Veterans Administration medical systems in 2010 (9).

The leading causes of TBI in the general population are falls (35.2%), motor vehicle crashes (17.3%), blunt impact (e.g., being struck by or against a moving or stationary object) (16.5%), and assaults (10%) (4). Different age groups are affected to varying degrees (Table). Falls account for a large proportion of TBIs among children aged 0–14 years and among adults aged ≥65 years (4). Motor vehicle crashes and assaults are the predominant causes of TBIs in teens and young adults aged 15–34 years (4). Military personnel, both in and out of combat, and rescue workers and victims exposed to blasts also are at risk for TBI (10).

TBIs can be categorized as mild (often called concussions), moderate, or severe based on the Glasgow Coma Scale (11). This and other categorization systems, although crucial for clinical management, generally do not reflect the underlying pathologic processes of the injury or nonfatal outcomes. The lack of a system for severity classification is one of the major gaps in the clinical assessment and treatment of TBIs (12,13).

Much of the brain injury occurs after the primary injury, not at the moment of initial impact. A complex biologic cascade begins immediately after the trauma and can continue for hours to weeks after the initial injury. It is this secondary injury that can significantly increase the overall morbidity and mortality that follows a TBI. Although research is ongoing, no drugs have yet been proven to reduce secondary injury and improve functional outcome of TBIs (14). The long-term or lifelong physical, cognitive, behavioral, and emotional consequences of a severe TBI can affect all aspects of a person’s life, including the ability to return to work or school and sustain relationships with family, friends, and community (2).

## Public Health Role in Addressing Severe TBIs — Challenges and Opportunities

Public health efforts coordinated across organizations and communities could help to reduce the incidence of TBIs and mitigate their short- and long-term consequences. Those efforts can include primary prevention, early management, and comprehensive approaches to rehabilitation and reintegration.

## Primary Prevention

Public health plays a key role in primary prevention of TBI by conducting surveillance, identifying and disseminating evidence-based strategies, and promoting implementation of effective policies. Several systems collect and report national and state-based TBI data used for surveillance, including multiple cause of death mortality data and vital statistics submitted to the National Vital Statistics System from all 50 states and the District of Columbia, basic TBI surveillance from the 20 states funded through the Core Violence and Injury Prevention Program, reports and data from the National Trauma Data Bank, and national estimates of injury-related emergency department visits from the National Electronic Injury Surveillance System.* These data collection tools are critical for monitoring TBI incidence and informing decision making on prevention initiatives, research needs, and education priorities. However, current data sources do not provide the level of detail needed to fully understand the epidemiology and long-term outcomes of TBI. A more comprehensive national injury surveillance system that enables population-based longitudinal or follow-up studies would better guide prevention efforts and aid in the evaluation of the effectiveness of interventions (2).

Public policies can advance prevention of TBIs and other injuries through education, enforcement of safety laws and regulations, engineering, and economic incentives. This is demonstrated by recent progress in reducing deaths and serious injuries from motor vehicle crashes. Since 1980, the rate of TBI-associated deaths caused by motor vehicle crashes decreased approximately 40%, in part because of a multitude of public policies and law enforcement. Those initiatives have included state laws and sustained, high-visibility enforcement that increased nationwide seatbelt use to 85% (15), universal motorcycle helmet laws in states that sustained helmet usage of 90% or higher (16), and enforcement of state laws lowering the legal limit for blood alcohol concentration to 0.08 g/dL and raising the minimum drinking age from 18 to 21 years (17). Despite these policy successes, ongoing challenges to injury and TBI prevention remain. In 2011, for example, alcohol-impaired driving still accounted for 31% of the total motor vehicle traffic fatalities in the United States (18).

Because the causes of TBIs vary among population groups, multiple educational and awareness efforts are needed to improve the primary prevention of severe TBI. For example, in the last decade, the number of children and adolescents who sought care in emergency departments for sports- and recreation-related TBIs, including concussions, increased 60% (19). In response, CDC, in collaboration with the National Collegiate Athletic Association, National Football League, and many associations governing sport activities, created concussion educational resources for coaches, athletes, and medical professionals.† To prevent fall-related TBIs among older adults, CDC partnered with stakeholder organizations to develop educational materials that describe evidence-based interventions to help public health practitioners, clinicians, community-based organizations, and older adults to prevent, recognize, and respond to the signs and symptoms of TBI.§ A leading cause of child maltreatment deaths in the United States, is “shaken baby syndrome” (abusive head trauma). The steps to implement evidence-based intervention strategies and integrate specific education messages into existing programs for new parents, caregivers, professionals, and the general public are outlined in the CDC publication, *Preventing Shaken Baby Syndrome: Guide for Health Departments and Community-Based Organizations*.¶

## Early Management

An effective public health response to TBI requires concerted programs to minimize adverse outcomes among injured persons, including efforts to improve acute care and early management, and strategies to ensure patient access to appropriate care and services. The CDC publication, *Guidelines for Field Triage of Injured Patients, Recommendations of the National Expert Panel on Field Triage, 2011*, was developed to help prehospital-care providers recognize injured patients who are most likely to benefit from specialized trauma center resources (20). The risk for death for a severely injured adult patient is 25% lower when the patient receives care at a Level I trauma center than at a nontrauma center (21). Unfortunately, nearly 45 million U.S. residents live more than an hour away from Level I or II trauma centers (i.e., hospitals that have the resources to treat patients with life-threatening injuries).

The Brain Trauma Foundation (BTF) guidelines for prehospital and in-hospital management of severe TBIs provide health-care professionals with evidence-based patient care and treatment recommendations.** A study assessing the effectiveness of adopting the BTF guidelines estimated that full implementation would result in a 50% decrease in deaths, a savings of approximately $288 million in medical and rehabilitation costs, and savings of approximately $3.8 billion in annual societal costs for severely injured persons who survived TBI (22). However, adherence to BTF guidelines in 2006 was approximately 65% in Level I and Level II trauma centers (23). Moreover, TBI mortality and morbidity vary widely, even in centers that report adoption of the guidelines.

The structure, organization, and use of emergency medical services and trauma systems can have a profound impact on improved acute care and early management of injured patients, the costs associated with trauma care, and on the lives of the millions of persons injured every year in the United States. Continued efforts are needed to promote widespread treatment guideline adoption, ensure early access to trauma care, and support the development of trauma systems that are integrated with public health systems across the United States. Ongoing collaboration among local, regional, and state emergency medical services agencies with governmental, nongovernmental, academic, and public health agencies and institutions will allow the continued analysis and evaluation of the effect of the guidelines on the care of acutely injured patients, including those with TBIs.

## Comprehensive Approaches to Rehabilitation and Reintegration

Because of the variability in how disabilities associated with TBI might permanently alter a person’s vocational aspirations and social and family relationships, each patient needs an individualized approach to rehabilitation and community reintegration. This ensures that each person reaches their maximum functional potential, learns to adapt to their disability, and maximizes the possibility that they will be able to return to their employment or former role in households and communities.

Current evidence shows that a comprehensive program of rehabilitation is the most effective way of helping patients regain function and minimize negative consequences of TBIs (24). Public health plays a critical role in supporting the rehabilitation and reintegration of patients into their communities and in identifying mechanisms for reimbursement that allow access to comprehensive care. Public health and the clinical community also need to collaborate to build the evidence base for effective strategies of comprehensive rehabilitation programs, disseminate best practices, and link rehabilitation care to public health interventions that support life-long health.

## Conclusions

TBI is an important public health problem that requires more attention, societal engagement, and research. The major aspects of public health interventions for TBI include primary prevention, early management, and comprehensive approaches to rehabilitation and community reintegration. TBIs can be prevented through available interventions, but those interventions must be implemented in coordination with commitment of multiple sectors of society, including efforts at federal, state, local and community levels. More research also is needed to understand the basic mechanisms and pathophysiology of TBI, and to identify treatments and therapies that can mitigate its long-term consequences.

## Figures and Tables

**TABLE:** Estimated average annual numbers and rates* of emergency department visits, hospitalizations, and deaths related to traumatic brain injury, by age group and external cause — United States, 2002–2006

Age group (yrs)	Motor vehicle crash		Falls		Assault		Blunt impact		Other/Unknown	
				
	No.	Rate	No.	Rate	No.	Rate	No.	Rate	No.	Rate
0–4	15,429	77.1	167,950	838.7	1,619	8.1	54,811	273.7	27,974	139.7
5–9	10,180	51.7	44,114	223.9	1,091	5.5	36,139	183.4	22,740	115.4
10–14	9,076	43.3	44,750	213.4	11,991	57.2	35,826	170.8	27,568	131.5
15–19	52,408	252.4	34,911	168.1	24,528	118.1	37,595	181.0	36,646	176.5
20–24	54,224	261.3	21,191	102.1	36,337	175.1	19,464	93.8	30,594	147.4
25–34	54,161	135.8	35,368	88.7	41,197	103.3	31,399	78.7	48,467	121.5
35–44	29,888	67.8	39,662	89.9	25,285	57.3	22,744	51.6	45,162	102.4
45–54	29,031	69.8	39,871	95.8	17,058	41.0	17,743	42.6	32,205	77.4
55–64	18,951	65.2	22,940	78.9	8,031	27.6	10,579	36.4	24,740	85.1
65–74	8,653	46.7	37,466	202.2	1,567	8.5	7,627	41.2	16,294	87.9
≥75	10,193	57.1	106,872	599.2	909	5.1	5,957	33.4	42,306	237.2

* Per 100,000 population.

## Footnotes

* Additional information available at http://www.cdc.gov/nchs/deaths.htm; http://www.cdc.gov/nchs/nvss.htm; http://www.cdc.gov/injury/stateprograms; http://www.facs.org/trauma/ntdb/index.html; and http://www.cpsc.gov/library/neiss.html.

† Available at http://www.cdc.gov/concussion.

§ Available at http://www.cdc.gov/homeandrecreationalsafety/falls/index.html.

¶ Available at http://www.cdc.gov/concussion/headsup/sbs.html.

** Guidelines for management of severe traumatic brain injuries are available from BTF at https://www.braintrauma.org/professional-homepage.
